# Climate and poverty in Africa South of the Sahara

**DOI:** 10.1016/j.worlddev.2019.104691

**Published:** 2020-01

**Authors:** Carlo Azzarri, Sara Signorelli

**Affiliations:** a International Food Policy Research Institute (IFPRI), 1201 Eye St, NW, Washington, DC 20005, USA; b Paris School of Economics, 48 Boulevard Jourdan, 75014 Paris, France

**Keywords:** Mapping, Poverty, Climate change, Weather shocks, Sub-Saharan Africa, Spatial models

## Abstract

To estimate the effects of weather conditions on welfare globally, cross-country comparisons need to rely on international poverty lines and comparable data sources at the micro-level. To this end, nationally representative household surveys can offer a useful instrument, also at the sub-national level. This study seeks to expand the existing knowledge on the determinants of poverty in Africa south of the Sahara (SSA), examining how long-term climatic conditions and year-specific weather shocks affect expenditure per capita. We take advantage of a novel and unique dataset combining consumption-based household surveys for 24 SSA countries -representative of more than half of the African population and two thirds of SSA- and geospatial information on agro-climatic conditions, market access and other spatial covariates of poverty. To our knowledge, it is the first time that a welfare-based, multidisciplinary, micro-level dataset with such wide spatial coverage has been assembled and examined. Our analysis relies on a linear and spatial model at the household- and district-level, respectively, both controlling for socio-economic, demographic, and geographic confounding factors. Results are consistent across econometric approaches, showing that living in more humid areas is positively associated with welfare, while the opposite occurs living in hotter areas, as existing literature shows. Flood shocks -defined as annual rainfall higher than one standard deviation from the 50-year average- are associated to a 35% decrease in total and food per-capita consumption and 17 percentage point increase in extreme poverty. On the other hand, extreme shortages of rain and heat shocks show an uncertain effect, even when estimates control for spatial correlation between welfare and weather conditions using the spatial error correction model. Given the heterogeneous effects of climatic events across SSA macro-regions, local-specific adaptation and mitigation strategies are suggested to help bringing households on a sustainable path.

## Highlights

• The study uses a novel, Africa-wide dataset of nationally-representative, consumption-based household surveys.
• In SSA long-term climate conditions yield differential effects on welfare compared to short-term climatic shocks.
• Excess rainfall disproportionately hinders welfare in selected SSA regions while drought shows uncertain spatial effects.
• Exposure to flood shock is associated to 35% decrease in consumption and 17 percentage points increase in extreme poverty.
• Smallholder farmers appear to be the most vulnerable to weather variability, both in case of floods and droughts.

## 1 Introduction

In the light of the global effort towards increasing welfare and sharing prosperity across different population groups, transcending national boundaries in the spatial analysis of poverty is becoming key. There is a growing need to identify major areas of indigence and to understand their determinants in order to properly address the root causes of poor welfare conditions. Whenever a specific poverty reduction intervention is under consideration, policy makers need to address two major questions: how many people will be lifted out of poverty and where are they located. Spatial targeting and mapping analysis can answer the second question, while the first one has to be addressed through multivariate statistical analysis using either experimental or non-experimental approaches.

Existing studies on international poverty are limited to country-level comparisons and, therefore, often overlook sub-regional heterogeneity, crucial for unpacking the spatial clustering of welfare. In addition, earlier literature attempting to link welfare with landscape-level conditions failed to account for spatial autocorrelation, thereby bringing into question the robustness of findings. The existing literature on the spatial dimension of poverty in an international setting mainly focuses on national-level poverty measures (Ferreira & Ravallion, 2008) and, in order to obtain national estimates comparable over time, combines national account data with welfare distributions drawn from household surveys, with the associated problematic assumptions of aggregation and comparability (Chen and Ravallion, 2014, Deaton, 2005, Edward and Sumner, 2014, Ferreira et al., 2015, Ravallion, 2003).

In addition, Sumner (2012) discusses how recent shifts in global poverty – with a greater number of poor living in middle income countries – reveal a change in the nature of poverty measurement, from cross-country differences in growth rates to intra-national welfare distributions. For the latter, looking beyond national-level welfare to focus on the distribution across regions and districts within countries becomes even more crucial. Sub-regional poverty analysis remains limited and mostly focused on single-country case studies (Amarasinghe et al., 2005, Bellon et al., 2005, Benson et al., 2005, Kam et al., 2005), or at best is separately conducted on different countries and subsequently compared across them (Hyman, Larrea, & Farrow, 2005) – an approach that is often dictated by the limited data comparability across household surveys (Beegle, Christiaensen, Dabalen, & Gaddis, 2016).

Sub-Saharan Africa (SSA) is one of the most vulnerable regions to climatic shocks, since it is home to the bulk of the world’s extreme poor who, given the projected demographic trends, are unlikely to escape extreme poverty in the near future (Beegle et al., 2016, Maddison et al., 2007, Winsemius et al., 2018), mostly due to the negative effects of climate change in African agriculture (Kempe, 2009, Morton, 2007, Muller et al., 2011). In addition, the World Bank estimates that a hundred million people globally are at risk of falling back into poverty as a direct result of climate change, with most affected households concentrated in South Asia and SSA (Hallegatte et al., 2016). Poor households are shown to live in the driest villages of dry areas, and in the wettest villages of wet areas, resulting in an inverted U-shaped relationship between (predicted) income and precipitation (Angelsen & Dokken, 2018). These findings underline the importance of understanding people’s vulnerability to climatic events in SSA across space.

The existing literature on the impacts of weather shocks on productivity, income and consumption is highly mixed, context-specific (Schlenker et al., 2005, Baylis et al., 2011 among others), and mostly focused on vulnerability. Little evidence exists on the specific impact of different types of weather shocks on household consumption (Narloch and Bangalore, 2018, Skoufias and Vinha, 2013). In this paper, we used an empirical approach similar to Deschênes and Greenstone (2007), although instead of using panel data we take advantage of cross-sectional data across a large number of countries and we look at the effect on aggregate welfare measures instead of agricultural income. We aim to answer two fundamental questions: 1) How are welfare and poverty distributed across Sub-Saharan Africa?; 2) What is the impact of long-term climatic conditions as well as extreme weather shocks on well-being, after controlling for potential confounding factors?

We propose an innovative analysis based on an internationally comparable sub-national consumption dataset for 24 SSA countries linked to location-specific agro-climatic characteristics. We rely uniquely on information from household surveys to construct our welfare measures, therefore avoiding the potential issue of micro-macro data comparability described by Ravallion (2003). In addition, due to our use of national purchasing power parity (PPP) conversion factors,^1^ our estimates are comparable cross-country. The computed sub-regional poverty measures allow to map different proxies of welfare conditions beyond country boundaries and, therefore, to look at the spatial distribution of welfare more closely. Finally, to explore the climatic determinants of well-being, we match district-level spatial information on biophysical characteristics -such as rainfall, temperature, drought index, and a measure of overall humidity- to the household dataset. To our knowledge, this is the first study that pulls together such a large collection of nationally-representative, multi-topic, consumption-based household survey data for SSA analyzed at the sub-national level. This large spatial and population coverage allows us to go beyond country-specific policy suggestions and draw some conclusions applicable to more than a third of the African population and half of the SSA region.

We also look at the determinants of poverty at the household and the sub-regional level across SSA, with a specific emphasis on the relationship between welfare and both long-term climatic conditions and year-specific weather events. Given that “small-scale and family farmers produce 80 percent of the food supply in sub-Saharan Africa and Asia” (FAO, 2017), we examine the effects mostly through the agricultural lens. Overall, results show that long-term agro-ecological conditions are associated the expected sign, with humidity positively correlated and temperature negatively correlated with welfare. Moreover, a negative and strong effect of excess rainfall on consumption and a positive association with extreme poverty is found regardless of the unit of analysis (household or district) and method (OLS or spatial models, respectively), while the effect of extreme drought is mixed.^2^ Estimates run by macro-regions (Eastern, Central, Western, and Southern Africa) show differential effects of climate events across agro-ecology, providing justification and rationale for local-specific strategies.

The rest of the paper is structured as follows. Section 2 describes the data; Section 3 presents the methodology; Section 4 discusses the results as well as the robustness checks; finally, Section 5 concludes.

## 2 The data

### 2.1 Household surveys

Our poverty calculations are based on the comparison between the household per-capita total consumption expenditure (a synthetic indicator expressing the money-metric welfare utility level) in the survey year and the $1.90 and $3.10/day per-capita poverty lines expressed in international equivalent PPP dollars in 2011. As such, poverty measures are computed in each survey year and then converted and discounted in value of the same reference year (2011), but are referred to sub-national relative distribution prevailing in the year of the survey. In this regard, cross-country sub-national poverty needs to be interpreted for a given (constant) purchasing power of each country's local currency compared to the international dollar benchmark.

The PPP factors bear a significant impact on the poverty measures obtained, and the release of the 2011 PPP conversion factors by the World Bank (2015) has significantly changed the relative poverty among countries from the previous estimates based on the 2005 PPP, although the overall trends of decreasing global poverty are confirmed (see Ferreira et al., 2015, Jolliffe, 2015 for a discussion on the importance of the PPP factors). Despite the PPP revisions and the general improvement in data availability for several African countries, “tracking poverty in Africa is difficult because the data are deficient on these three domains: availability, comparability, and quality” (Beegle et al., 2016).

Our poverty estimates are based uniquely on household survey information and thus avoid possibly problematic issues arising with methods combining income per capita growth from national accounts and welfare distribution from micro-data (Chen and Ravallion, 2014, Deaton, 2005, Ravallion, 2003). In addition, this strategy allows us to compute consistent values of subnational poverty headcount using survey’s expansion factors,^3^ which guarantee the statistical validity and representativeness of the estimates at the stratum at which each survey is representative, and to render the measures comparable across countries by using the PPP adjustments. The results obtained at the national level are compared to the statistics in the World Bank PovcalNet database^4^ to ensure consistency to the World Development Indicators.^5^ Table 1 presents the basic information of each household survey included in our dataset. Survey year ranges from 2002 (Lesotho) to 2014 (Mali and Burkina Faso).

**Table 1:** Household datasets used.

Country	Dataset code and year	Admin. level units	Admin. level 2 units	N. of household observations	Population in survey year (Mio. People)
Angola	IBEP – 2008	18	80	7249	16.4
Burkina Faso	EMC – 2014	13	30	10,411	1.7
Burundi	CWIQ – 2006	16	113	6489	8.1
Cameroon	ECAM – 2007	10	56	9320	17.9
Cote d'Ivoire	ENV – 2008	10	–	12,600	17.6
Congo, DRC	123–2012	25	–	19,270	79.1
Ethiopia	HCES – 2010	11	474	27,835	76.1
Ghana	GLSS – 2012	10	163	14,812	26.3
Kenya	IHBS – 2005	8	67	11,976	34.6
Lesotho	HBS – 2002	10	–	5992	1.7
Madagascar	EPM – 2010	22	–	12,460	18.8
Malawi	HIS – 2011	8	27	12,271	14.0
Mali	EMOP – 2014	7	43	5227	17.1
Mauritania	EPCV – 2000	12	39	4255	2.0
Mozambique	IOF – 2008	10	126	10,832	21.5
Niger	ECVMA – 2011	7	33	3859	16.5
Nigeria	GHS – 2012	6	37	4536	174.0
Rwanda	EIC – 2005	12	28	6900	9.5
Senegal	ESPS – 2011	14	42	5953	13.6
South Africa	IES – 2011	9	–	25,328	50.4
South Sudan	NBHS – 2009	10	–	4969	8.4
Tanzania	NPS – 2012	8	25	4883	45.1
Uganda	NHS – 2013	4	110	6886	34.0
Zambia	LCMS – 2010	9	71	19,389	11.3

For most surveys, information on administrative level-2 (commonly referred as district) is available, and hence this is also the level at which spatial information has been matched.^6^ For the few countries where district information is not available, spatial variables have been matched at the administrative level-1 (usually, the region). Countries in all regions of SSA are represented, providing a balanced sample across different agro-ecologies. In addition to our main welfare measures of interest, we rely on household survey data for several socio-demographic characteristics used as control variables in the regressions, such as household gender and age composition, and education.

### 2.2 Spatial data

To capture long-term climatic conditions as well as year-specific extreme weather shocks, we use sub-regional monthly data on rainfall and temperature (Willmott & Matsuura, 2017), as well as the Standardized Precipitation Evapotranspiration Index (SPEI) index of evapotranspiration (Beguería and Vicente-Serrano, 2016, Vicente-Serrano et al., 2010).^7^ The SPEI is an extension of the Standardized Precipitation Index, which takes into account precipitation, temperature, and potential evapotranspiration in determining drought. Given the scarce use of irrigation in most of SSA, Liebmann et al. (2012) claim that the rain patterns during planting and growing seasons are the most critical to determine farmers’ livelihoods. We therefore refer to the FAO maize cropping calendar to identify the months of cropping and growing season for each country in our sample,^8^ over which we construct annual and long-term averages for the spatial variables of interest. Our measure of weather shock is a dummy taking the value of one if the year-specific weather value (limited to the months of planting and growing season) is higher (lower) than + 1 (-1) or + 2 (-2) standard deviations (SD) from the long-term average, the latter computed from the 1950s up until the year of the survey for the same months of planting and growing seasons, and for each country and region. Given the fact that household interviews in the dataset were conducted at different times of the year, as such they are sometimes conducted before the growing season and sometimes after it. Since our aim is to measure the effect of weather shocks happened during the cropping season preceding the interview, which are expected to be the most detrimental for agricultural output, we match weather information from the year prior to the survey data collection (for each country/survey) for households interviewed before the end of the growing season, while we use contemporaneous weather data for households interviewed after the beginning of the harvest. Finally, to ensure that results do not depend on the choice of the shock measure considered, we test two alternative measures of shock computed using: 1. long-term average from 1990 onwards; and 2. long-term median -instead of the average- since 1950.

**Fig. 1:**
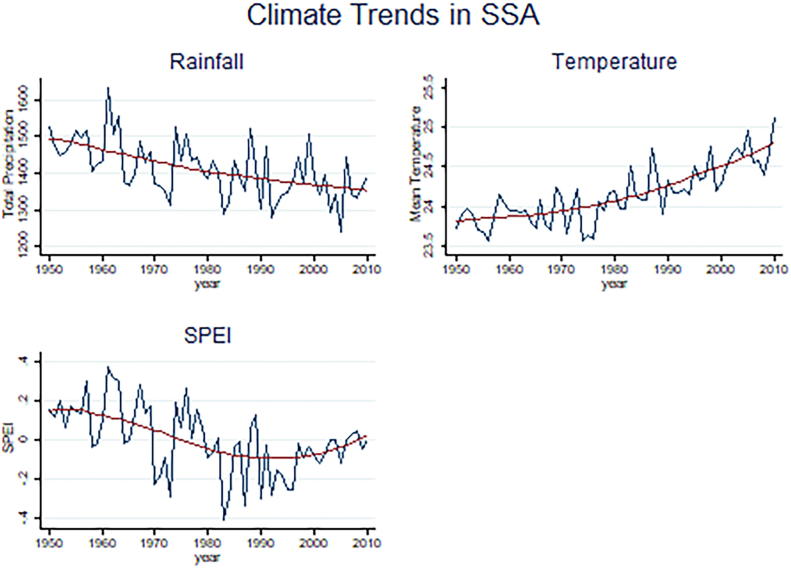
Climatic Trends in SSA. *Note: trendsare weighted by district areas in Km.*

Fig. 1 reports the main climatic trends observed across SSA. Rainfall has been steadily decreasing while the opposite has happened for temperature. In terms of evapotranspiration, proxied by the SPEI, a downward trend until the mid-1990s and then a slight increase, and especially a decrease in overall volatility, can be observed. Appendix A reports climate trends disaggregated by region.

In addition to weather measures, we also compute additional sub-regional level variables derived from remote sensing data in order to control for other potential factors confounding the relation between climate variability and welfare. We condition our estimates by population density and infrastructure development, proxied by the index of nightlight; agricultural potential, proxied by the length of the growing period; elevation; per capita cropland area; per capita tropical livestock units (Koo et al., 2016); average travel time from a market (>20 k population); and finally, for sensitivity estimates reported in the appendix, climate-related morbidity proxied by malaria incidence (Bhatt et al., 2015, Frank et al., 2003, Kibret et al., 2016, Paaijmans et al., 2009, Rogers and Randolph, 2000, Utzinger et al., 2005).

## 3 Methodology

Our preferred outcome variables of interest are the logarithm of household per-capita total and food consumption expenditure as well as poverty rate computed using the threshold of 1.90$/day. Based on the literature on the determinants of welfare and poverty (Dercon and Krishnan, 2000, Benson et al., 2005, Banerjee and Duflo, 2007 among others) we consider the following model:

$$Y_{k}=\text{F}\left(D,A,E,I,S,C\right)$$

where *Y*, measuring the welfare of a household (*h*) for *k = h* and the average welfare of a district (*d*) for *k = d*, is a function of socio-demographic characteristics (*D*), agricultural production potential (*A*), environmental conditions including climate and altitude (*E*), access to infrastructure (*I*), year-specific weather shocks (*S*) and country-specific fixed effects (*C*). We estimate a simple pooled-OLS model as follows:

$$Y_{\mathrm{cdht}}=\alpha +\beta_{1}D_{\mathrm{cdht}}+\beta_{2}A_{\mathrm{cdt}}+\beta_{3}E_{\mathrm{cd}}+\beta_{4}I_{\mathrm{cdt}}+\beta_{5}S_{\mathrm{cdt}-1}+\gamma_{c}+\tau_{t}+\delta_{m}+\varepsilon_{\mathrm{cd}ht}$$

Where, depending on the specification used, $Y_{\mathrm{cdht}}$ expresses 1. the logarithm of household per capita total expenditure and food expenditure in international $ (2011 PPP) or 2. the indicator for extreme poverty (1.90$/day) for country *c*, district *d*, household *h* and time *t*.^9^ All the controls are computed for the survey year, except for the long-term climatic conditions and the weather shocks, as reported above. Country fixed effects ($\gamma_{c}$) are included to control for unobservable characteristics such as differences in institutions, rule of law, and idiosyncratic shocks that may have occurred in specific countries. Year fixed effects ($\tau_{t}$) and month fixed effects ($\delta_{m}$) are included to control for time-varying shocks and for seasonality.

Moran’s I test statistic, Lagrangian multiplier, and robust Lagrangian multiplier tests are conducted to assess the spatial correlation between weather variables and outcome measures at the district level. Since covariates in our model can be spatially correlated, as can be the error term in the presence of omitted spatial variables, we estimate the district level model using spatial regression analysis (Anselin, 2002, Anselin et al., 2004, Paraguas and Kamil, 2005). We construct a spatial weighting matrix defining the neighborhood of influence of each district through the inverse of the distance from each district centroid. Three models can be used to estimate spatial correlation: a spatial lag model, a spatial error model, and a spatial auto-correlation model (SAC), with the last being a combination of the first two. The first introduces a weighted mean of the outcome variable in neighboring districts as additional regressor (*W*), as follows:

$$Y_{\mathrm{cdt}}=\alpha +\rho W_{y}+\beta_{1}D_{\mathrm{cdt}}+\beta_{2}A_{\mathrm{cdt}}+\beta_{3}E_{\mathrm{cd}}+\beta_{4}I_{\mathrm{cdt}}+\beta_{5}S_{\mathrm{dt}-1}+\gamma_{c}+\varepsilon_{\mathrm{cdt}}$$

The second model estimates spatial dependence in the error term, according to:

$$Y_{\mathrm{cdt}}=\alpha +\beta_{1}D_{\mathrm{cdt}}+\beta_{2}A_{\mathrm{cdt}}+\beta_{3}E_{\mathrm{cd}}+\beta_{4}I_{\mathrm{cdt}}+\beta_{5}S_{\mathrm{dt}-1}+\gamma_{c}+\varepsilon_{\mathrm{cdt}},\;{where\;\varepsilon }_{\mathrm{cdt}}=\lambda W_{\varepsilon }+\varepsilon_{\mathrm{cdt}}$$

We use the Lagrangian Multiplier test on ρ and λ to test their significance and, hence, choose the appropriate model. According to the test results, regressions are consistently estimated through the SAC model, which accounts for correlation both in the outcome variable and in the error term (ρ ≠ 0 and λ ≠ 0). Through this method we can assess the major determinants of district welfare across SSA and test the specific contribution of climatic characteristics.

Finally, to test how the impact of weather shocks differs across farming and non-farming households, we include interaction terms between the shock measures and the farming status of the household:

$$Y_{\mathrm{cdht}}=\alpha +\beta_{1}D_{\mathrm{cdht}}+\beta_{2}A_{\mathrm{cdt}}+\beta_{3}E_{\mathrm{cd}}+\beta_{4}I_{\mathrm{cdt}}+\beta_{5}S_{\mathrm{cdt}-1}+\beta_{6}FH_{\mathrm{cdht}}+\beta_{7}S_{\mathrm{cdt}-1}*FH_{\mathrm{cdht}}+\gamma_{c}+\tau_{t}+\delta_{m}+\varepsilon_{\mathrm{cd}ht}$$

Where $FH_{\mathrm{cdht}}$ includes a dummy for smallholder farmers (<2 ha of land) and a dummy for large landowners (>2 ha), with non-farming households as reference category. The $\beta_{7}$ parameter estimates capture the differential effect of weather shocks on these two groups relative to non-farmers.

## 4 Results

### 4.1 Summary statistics

Table 2 shows the summary statistics of the main outcome variables. According to our estimates, the poorest countries in our sample seem to be Madagascar, Burundi, and Congo DRC, with overall poverty rates close to 90% and extreme poverty rates above 75%. On the other hand, the better-off countries seem to be Ghana and South Africa, both with <15% of extremely poor population.

**Table 2:** Poverty rates for different measures.

Country	Expenditure per capita (2011 PPP$)	Poverty headcount ratio ($1.90 2011PPP)	Poverty headcount ratio ($3.10 2011PPP)	Food share of consumption	Food expenditure per capita (2011 PPP $)	Food Poverty (headcount bottom quintile)	Food Poverty headcount (bottom 2 quintiles)
Angola	120.7	30%	55%	58%	61.0	7%	21%
Burkina_Faso	83.8	44%	75%	53%	42.6	11%	42%
Burundi	47.6	77%	92%	69%	31.9	29%	62%
Cameroon	124.2	29%	54%	–	–	–	–
Cote d'Ivoire	130.0	24%	51%	47%	56.5	14%	31%
Congo, DRC	47.1	76%	90%	68%	29.6	37%	63%
Ethiopia	83.3	37%	74%	52%	39.9	14%	36%
Ghana	191.0	12%	31%	55%	94.6	3%	10%
Kenya	101.6	43%	67%	63%	51.2	12%	31%
Lesotho	69.4	62%	79%	–	–	–	–
Madagascar	43.9	82%	93%	71%	28.7	35%	66%
Malawi	59.4	68%	87%	63%	33.7	33%	58%
Mali	76.5	49%	78%	–	–	–	–
Mauritania	138.1	20%	44%	55%	70.1	7%	18%
Mozambique	58.9	68%	88%	63%	33.3	32%	56%
Niger	81.1	37%	75%	69%	52.7	2%	14%
Nigeria	70.3	55%	76%	73%	48.9	21%	40%
Rwanda	71.6	67%	84%	62%	38.0	30%	58%
Senegal	96.0	38%	66%	57%	48.6	11%	28%
South Africa	399.3	14%	32%	26%	51.5	21%	39%
South Sudan	96.8	43%	64%	79%	76.0	14%	26%
Tanzania	95.7	42%	67%	74%	64.3	7%	20%
Uganda	103.8	34%	64%	–	–	–	–
Zambia	81.2	60%	76%	58%	39.0	36%	57%

The country ranking is similar when looking at poverty based on food expenditure, which is highly and positively correlated with the share of food over total consumption expenditure. However, we find cases such as South Sudan, with a very high food consumption share (79%), but a mid-range (43%) extreme poverty headcount ratio. The summary statistics of the main controls at the household- and district-level can be found in Appendix B (Table B1, Table B2), in addition to the percent value distribution of the main weather shocks above 1 or 2 standard deviation from the long-term average (Table B.3)

### 4.2 Spatial distribution of poverty and climate

**Fig. 2:**
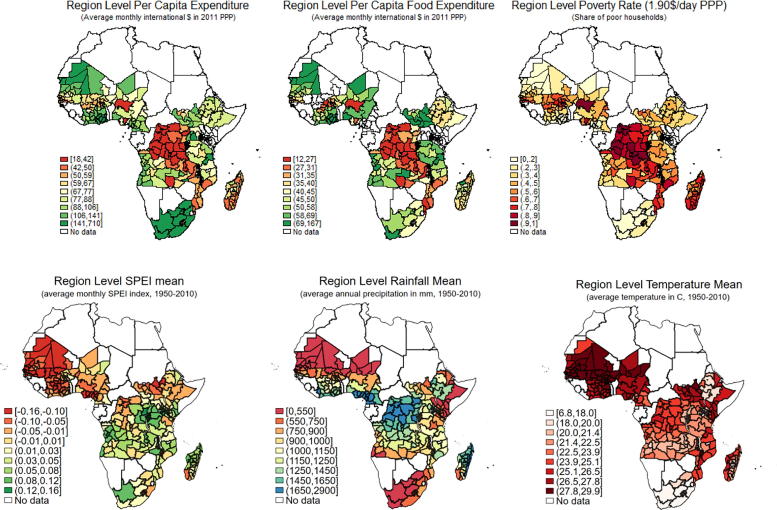
Distribution of welfare and climate across regions.

Poverty in SSA appears to be spatially clustered in specific sub-regions (in addition to the most common concentration in definite countries). As shown in Fig. 2, high poverty rates can be found in Congo DRC in Central; some areas in Tanzania, Zambia, and Malawi in Eastern; Madagascar in Southern; Nigeria and Niger, and some areas in Northern Ghana in Western Africa.^10^ The areas with the highest poverty in Eastern and Central Africa are also the most humid, as it can be seen from the rainfall map. Both household per-capita total and food consumption expenditure within countries are associated to higher spatial intra-cluster correlation the smaller the area, signaling a poverty prevalence in neighboring districts greater than in more distant districts (Appendix B, Table B.4). Similarly, the spatial correlation based on the inverse distance weighting matrix proxied by the Moran I statistics (shown in Appendix B, Table B.5) confirms the presence of statistically significant spatial dependency and, therefore, justifies the use of spatial regression analysis. The descriptive evidence suggests that welfare and climatic conditions are likely to be correlated, providing justification for a multivariate regression analysis to examine the relative importance of the determinants affecting welfare conditions, controlling for possible confounding factors.

### 4.3 Multivariate analysis

We ran separate specifications for the climate and shocks variables defined using SPEI, temperature, and rainfall, both using pooled OLS (for household-level data) and spatial autocorrelation model (for district-level data). Appendix Table B.6 reports the list of countries included in the regressions on per capita total expenditure and poverty headcount ratio (all 24 countries); and in the regressions on per capita food expenditure (only 20 countries due to data limitations).

Table 3 reports a summary of the main parameters of interest from the household-level regression models obtained using different climatic variables and based on the more complete specifications where country, year, and month fixed effects, as well as demographic and locational variables, are included as controls. Regressions are weighted by population in each country, with standard errors clustered at the regional level. A higher level of SPEI indicates higher moisture, beneficial for the crop growth and, therefore, positively correlated to agricultural productivity and production. Results show that in humid areas total and food expenditure are higher than in drier areas and, consequently, extreme poverty is lower. Even more interesting is that a flood shock -an extreme weather event identified by an annual value of SPEI higher than 1 SD from the average long-term SPEI- bears high and strong negative effect on welfare, while the effect of a drought shock -an extreme weather event identified by a value of SPEI lower than 1 SD from the average long-term SPEI- is not statistically significant using OLS (that is when estimates do not control for spatial correlation).

**Table 3:** Main household-level regression results.

	Log per capita expenditure ($ 2011 PPP)	Food per capita expenditure ($ 2011 PPP)	Poverty Rate 1.90$/day ($ 2011 PPP)
	(1)	(2)	(3)
	coef/se	coef/se	coef/se
*Panel A: Using SPEI shocks*			
SPEI, long term average	1.222***	1.374***	−0.596***
	(0.141)	(0.143)	(0.075)
SPEI flood shock dummy (1 sd)	−0.195***	−0.185***	0.134***
	(0.057)	(0.059)	(0.031)
SPEI drought shock dummy (1 sd)	0.060	0.026	−0.038
	(0.050)	(0.054)	(0.031)

Controls	ALL	ALL	ALL
Number of observations	2,49,876	2,19,033	2,50,000
N_pop	69,97,91,188.413	62,86,82,773.442	69,99,39,086.214
N_strata	24.000	20.000	24.000
R2	0.470	0.267	0.281

*Panel B: Using temperature and rain shocks*			
Rainfall long term average (dm)	−0.001	0.000	−0.001**
	(0.001)	(0.001)	(0.000)
Temperature long term average (C)	−0.046***	−0.046***	0.028***
	(0.006)	(0.006)	(0.003)
Rainfall flood shock dummy (1 sd)	−0.354***	−0.340***	0.167***
	(0.047)	(0.056)	(0.026)
Rainfall drought shock dummy (1 sd)	0.164***	0.167***	−0.103***
	(0.040)	(0.051)	(0.023)
Temperature heat shock dummy (2 sd)	0.124*	0.135*	−0.012
	(0.065)	(0.071)	(0.038)

Controls	ALL	ALL	ALL
Number of observations	2,50,552	2,19,692	2,50,676
N_pop	70,12,73,629	63,00,51,532	70,14,21,527
N_strata	24	20	24
R2	0.475	0.271	0.285

Note: 0.01 – ***; 0.05 – **; 0.1 – *.

Standard errors clustered at region level. Country, year, and month fixed effects as well as socio-demographic and geograhic controls included but not reported. Panel A identifies shocks using SPEI while Panel B identifies shocks using temperature and rainfall. SPEI is an index of evapotranspiration that combines temperature and humidity, with a high level of SPEI identifying hot and humid areas. Flood shock is a dummy that equals one if the value of SPEI for the planting and growing season preceding the survey is above 1 SD from the long term average (for the same months in the same region). Drought shocks are identified the same way except that the value is higher than 1 sd below the long term average. In Panel B shocks are identified using information on rainfall and temperature. Here the flood/drought dummy equal 1 when rainfall in the previous season is 1 SD above/below the long term average, while heat shock equal 1 when the temperature is 2 SD above the long term average. Countries included in regressions on per-capita total expenditure and on poverty rate are Angola, Burundi, Burkina Faso, Cote d'Ivoire, Cameroon, DRC, Ethiopia, Ghana, Kenya, Lesotho, Madagascar, Mali, Mozambique, Mauritania, Malawi, Niger, Nigeria, Rwanda, Senegal, South Sudan, Tanzania, Uganda, South Africa and Zambia. Regressions on per-capita food expenditure exclude Uganda, Mali, Lesotho, and Cameroon because of lack of officially calculated food data from the household surveys.

Long term rainfall average is not significantly correlated with welfare while long-term temperature is negatively and statistically correlated. Flood shocks are significantly welfare-decreasing (consistently with SPEI), while extreme shortages of rain and excess heat show an uncertain effect. This finding could signal a lower household resilience to flood than drought -especially in drier areas where soil is less able to retain water-, perhaps due to the diffusion of drought-tolerant varieties of main crops and the greater adaptation of farming systems to rainfall shortage in several SSA countries.

Although regressions are based on a cross-sectional -and not panel- dataset, the explanatory power of the models is remarkable, with a R^2^ around 47% for the models on per capita total expenditure and around 27–28% for those on per capita food expenditure and probability of being poor. The underlying represented population is almost 70% of the one billion people living in SSA, and more than half of the entire African population.

Appendix C.1 reports the complete set of OLS coefficients for the most parsimonious and the least parsimonious specifications when the SPEI indicator is used. All control variables show the expected sign, with parameters of the variables of interest stable across specifications. Female- and younger-headed households are associated with lower consumption level than the rest of the households. On the contrary, education level is positively associated with welfare, in line with the literature (Banerjee and Duflo, 2007, Benson et al., 2005, Wollard and Klasen, 2007). For locational factors, population density (proxied by nightlight), elevation, and per-capita land size are significantly and positively associated with welfare.

Appendix C.2 tests the effect of including an asset index as a control in the regression.^11^ Given that this measure cannot be constructed for Ethiopia due to data limitations, our estimates are based on the remaining 23 countries. Results show that the effect of long-term climatic conditions is unchanged, while the negative impact of floods is slightly smaller once we control for assets. Given the coefficients of interests are similar in magnitude, we decide to exclude this control in order to maximize the number of countries in our regressions. Appendix C.3 shows the robustness of the coefficients to the inclusion of an indicator for malaria incidence, showing that both magnitude and sign of the parameters of the main outcomes of interest remain unchanged.^12^

Appendix C.4 shows regression results for the subsample of 20 countries for which data on all the three outcomes of interest -including food expenditure- are available, run both on the overall sample and on the rural sample only. The negative impact of floods is robust regardless of the outcome variable and the sample considered. Long-term SPEI has a stronger positive effect in rural areas while a drought -measured through extremely low rainfall- shows overall a positive coefficient that becomes smaller and eventually loses statistical significance when only rural areas are considered.

Appendix C.5 tests the robustness of the results to alternative measures of long-term climatic conditions and shocks. Regardless of the climate variable considered, living in more humid areas is positively associated with welfare, while the opposite occurs in hotter areas. However, flood shocks are significantly welfare-decreasing, while extreme shortage of rain and excess heat show an uncertain effect, controlling for various observable confounding factors at the household-level.

**Fig. 3:**
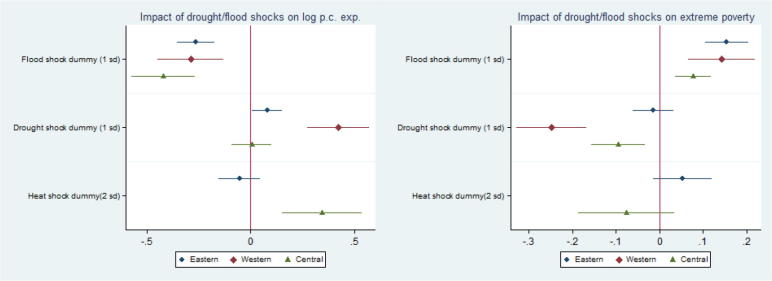
Summary of OLS main parameters of interest by region.

Looking beyond SSA average effects, Fig. 3 shows the main household-level OLS parameters of interest separately by region.^13^ Results show that floods are detrimental across all regions while droughts yield differential effects across region and agro-ecology, with Western Africa showing positive effects associated with shortage of rain, while no significant effect is found in the other regions. Finally, heat shocks seem to be beneficial in Central Africa.

Results at the household-level are confirmed by district-level regressions (in Appendix D), for which the spatial autocorrelation model is estimated given the -econometrically tested- presence of spatial correlation between weather variables and welfare measures. Once all available observable characteristics are controlled for, climatic variables in the district spatial-error models show parameters’ sign for long-term averages and year-specific shocks similar to the household-level regressions’, for both flood and drought shocks (Appendix D.1). Regardless of the econometrics model used, the effect of a flood remains negative and strongly statistically significant, while the one associated to drought remains uncertain (Appendix D.2). This result is in line with recent literature, pointing to a disproportionate exposure of poor people to floods, especially in African urban areas (Winsemius et al., 2018). Long-term temperature shows a negative impact on household per capita total and food expenditure, and a positive effect on poverty rates, consistently with the household regressions. Finally, effects of heat waves remain uncertain.

To assess the heterogeneity of the effects between farming and non-farming households, Appendix E reports the results from household-level regressions with the inclusion of dummies identifying smallholder and large-holder farmers -defined using the 2 ha threshold-, further interacted with the SPEI -based shocks. Unfortunately, not all the surveys in our sample include information on land size (see Appendix B, Table B.6 for details). However, our sampled farmers show lower consumption per capita -and consequently higher poverty rates- compared to non-farming households, especially smallholders, who seem to be the poorest. Floods negatively affect farmers, again more disproportionately smallholders, with the latter associated to roughly 20% drop in per capita expenditure and 15 percentage points surge in extreme poverty. These findings confirm that smallholder farmers are the most vulnerable to climatic variability.

Finally, we use our district-level estimates to simulate the impact of the expected climate change on the level and spatial distribution of poverty. District-specific temperature and rainfall linear trends observed over the 10 years preceding the survey are extrapolated to predict expected temperature and rainfall over 5- and 10-year time horizon, under the assumption of linear patterns. Predicted future rainfall and temperature are then used to compute expected flood, drought, and heat shocks defined as before -that is, based on 1 or 2 SD difference from long term average-.

**Fig. 4:**
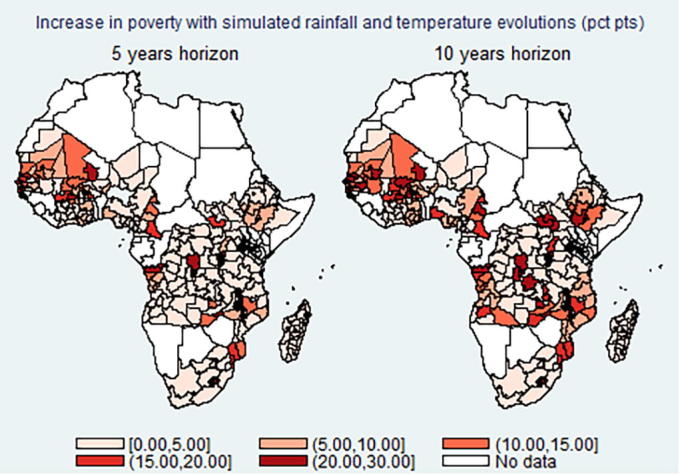
Simulated increase in shocks and expected effect on poverty by region.

Table 4 reports the simulated changes in rainfall and temperature with the associated percent changes in incidence of flood, drought, and heat shocks. On average, both rainfall and temperature are expected to increase the longer the time horizon considered, although with substantial heterogeneity across districts: 40% of districts will experience an expected decrease in rain and 30% of them an expected decrease in temperature. The standard deviation of rainfall and temperature across districts is expected to increase (in line with the literature, see Collier et al., 2008, Hulme et al., 2001 among others). In turn, the incidence of shocks is also expected to increase dramatically, with floods, droughts, and heat waves affecting about 20% of the districts in the 5- and 10-year horizon. We use the coefficients obtained from the main district-level regressions (see Appendix D) to simulate the predicted increase in district-level poverty rates, applying them to the expected future incidence of shocks, keeping all other variables constant. Fig. 4 maps the predicted difference in extreme poverty rates using the two climate horizons (5 and 10 years). These results should be taken cautiously, as they are based on some general assumptions such as no improvements in population adaptive capacity and no changes in the underlying consumption distribution, although we believe they provide a useful picture of the worst-case scenario under current conditions, i.e. if no further efforts are undertaken.

**Table 4:** Simulated future changes in rainfall and temperature.

Variable	Actual	5 years horizon	10 years horizon
Rainfall (mm)	454	456	458
Rainfall standard deviation (mm)	705	714	728
Temperature (C)	23.8	23.9	24.0
Temperature standard deviation (C)	4.1	4.0	4.0
Flood shock (% districts)	3%	14%	26%
Drought shock (% districts)	4%	15%	22%
Heat shock (% districts)	2%	12%	19%

Note: We simulate future rainfall and temperature at the district-level and at the 5 and 10 years horizon by calculating the district-specific linear time trend in rainfall and temperature observed over the 10 years preceding the survey, and extrapolating it forward. We then recompute the flood/drought shock equal 1 when predicted future rainfall is 1 SD above/below the long term average, while heat shock equal 1 when predicted future temperature is 2 SD above the long term average.

The areas expected to be hit the hardest by climate change are Western and Central Africa, with some areas projected to suffer an increase in extreme poverty up to 30 percentage points. This picture clearly shows the urgency to reinforce the resilience capacity of the most vulnerable populations, especially smallholder farmers, and to enact locally-specific interventions.

## 5 Conclusions

Our study is based on a large collection of recent nationally-representative household survey data for 24 countries in Sub-Saharan Africa, allowing comparison of descriptive statistics as well as inference for more than half of the African population and more than two thirds of SSA. Our database includes various welfare, consumption-based measures traditionally used to contrast poverty and inequality across countries, with the advantage of the sub-national breakdown. Surveys are centered around the year 2008, although monetary values have been expressed in 2011 purchasing power parity, allowing comparison of household samples taken at different points in time. Both per capita total and food consumption expenditure within countries are associated to higher spatial intra-cluster correlation the smaller the area, signaling a poverty concentration among neighboring districts greater than among distant districts.

An important feature of our study is the use of various long-term weather characteristics, matched with household survey data. On average rainfall has been falling since the 1950s while temperature increasing. Evapotranspiration has followed a downward trend until the mid-1990s, after which it has experienced a slight increase and especially a decrease in volatility. Biophysical variables show differential patterns across SSA regions.; while temperature has steadily increased in the whole continent, rainfall has decreased disproportionately in Western Africa.

Using our large household survey dataset, matched with spatial biophysical data, we provide estimates of the effects of long-term climate characteristics as well as short-term extreme shocks on welfare conditions proxied by total and food consumption expenditures in SSA. Our analysis points to a statistically significantly positive (negative) association with long-term humidity (temperature): based on the household-level regressions, one additional standard deviation in SPEI long term average is associated to 11% higher per capita consumption while one additional degree Celsius is associated to 4.6% lower per capita consumption expenditure and 2.8 percentage points increase in poverty rates. Flood shocks, regardless of the measures considered, yield negative impacts on total expenditure. Indeed, being exposed to a flood shock -defined as an annual rainfall higher than one standard deviation from the 50-year average- is associated to a 35% decrease in total and food per-capita consumption and 17 percentage point increase in extreme poverty. On the other hand, a drought shock shows ambiguous effects, even when estimates control for spatial correlation between welfare and weather conditions. These findings are markedly different by region: while floods are associated to detrimental effects across all Sub-Saharan Africa, droughts are associated to better outcomes in West Africa and heat waves are associated with improved outcomes in Central Africa.

The analysis at the district-level, where the spatial autocorrelation model is used to control for spatial correlation between climate characteristics -both long- and short-term- and welfare outcomes, confirms the conclusions based on the household-level analysis, even with an increase in magnitude. Using the district-level spatial model, flood shock is associated to about 50% to 60% decrease in per-capita total and food consumption and 27 percentage points increase in poverty headcount ratios. Smallholder farmers appear to be the most vulnerable to weather variability, both for floods and droughts, compared to both large-holder farmers and non-farming households. Finally, our simulated future increase in incidence of weather shocks shows alarming effects on regional poverty rates as a result of climate change, if resilience capacity of vulnerable populations remains unchanged. This finding is especially important from a policy perspective, although other mechanisms could be tested using the novel database available from this study opening many other avenues of further important research on the poverty-weather nexus as well as resulting policy options and responses.

## Figures and Tables

**Figure:**
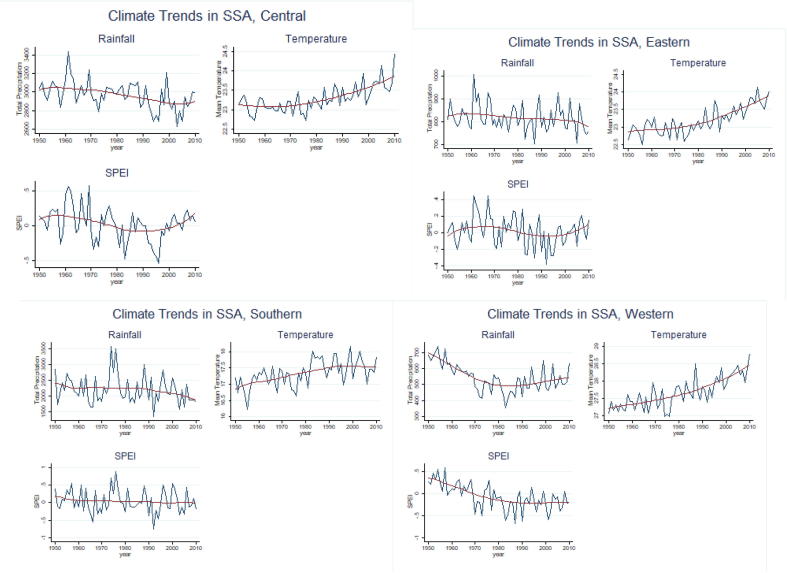


**Table B1:** Summary main controls – household survey variables.

ISO3	Area is rural	Household head is Female	Age of household head	The household head is married	Mean level of educ of adults (>15) in the household	Dep. Ratio (working age/hhsize)	Number of children (<6) in the household
Angola	55%	18%	42.2	16%	4.3	49%	1.48
Burundi	94%	15%	44.0	82%	3.7	49%	1.24
Burkina Faso	90%	5%	46.7	83%	2.1	48%	1.47
Cote d'Ivoire	59%	16%	45.5		3.7	57%	1.29
Cameroon	65%	21%	44.4	73%	6.1	90%	3.37
DRC	61%	16%	44.7	78%	6.6	52%	1.43
Ethiopia	83%	18%	44.2	84%	2.5	50%	1.13
Ghana	50%	25%	46.8	68%	7.1	56%	0.95
Kenya	79%	26%	46.0	80%	6.6	61%	1.18
Lesotho	76%	30%	50.8	64%	2.2	58%	0.81
Madagascar	78%	14%	43.4	77%	4.6	51%	1.17
Mali	76%	5%	53.2	94%	2.1	48%	2.97
Mozambique	70%	24%	42.6	82%	3.3	48%	1.36
Mauritania	60%	17%	49.9	100%	2.4	54%	1.52
Malawi	85%	20%	42.5	81%	5.4	50%	1.13
Niger	83%	7%	46.7	93%	2.7	44%	2.24
Nigeria	63%	10%	51.3	88%	6.8	50%	1.17
Rwanda	83%	24%	45.1	61%	3.7	53%	1.18
Senegal	57%	24%	54.2	86%	3.3	53%	2.56
South Sudan	84%	29%	43.6	92%	2.7	49%	1.67
Tanzania	74%	22%	47.0	67%	5.6	53%	1.28
Uganda	77%	27%	43.8	80%	5.6	49%	0.99
South Africa	39%	43%	48.9		9.4	64%	0.79
Zambia	61%	20%	43.4	79%	6.3	63%	0.31

**Table B2:** Summary of main controls – spatial variables.

ISO3	Nighlight radiation	Growing period (days, mean)	Elevation (meter)	Cropland pcap (Ha)	TLU pcap	Malaria Incidence (%)	Time to 20 k market (median hrs)	SPEI index ave. (1950-sv. year)	Rainfall ave. (1950-sv. Year, mm)	Temperature ave. (1950-sv. Year, C)
Angola	11.7	152	768	0.21	0.20	10%	4.70	0.08	280.9	23.5
Burundi	1.2	268	1622	0.17	0.07	22%	1.83	0.08	195.2	19.9
Burkina Faso	0.7	141	305	2.85	4.64	40%	2.93	−0.06	304.3	29.9
Cote d'Ivoire	10.9	287	200	0.39	0.10	64%	3.49	−0.03	1465.1	26.7
Cameroon	4.1	249	652	0.39	0.31	42%	3.20	−0.02	446.6	25.2
DRC	0.3	285	769	0.09	0.02	36%	10.53	0.01	2555.7	22.9
Ethiopia	2.3	203	1903	0.15	0.34	0%	3.73	−0.06	313.8	19.4
Ghana	12.0	261	167	0.26	0.08	36%	3.79	0.00	310.5	26.5
Kenya	2.8	236	1459	0.16	0.34	12%	1.89	0.01	293.4	19.8
Lesotho	0.6	178	2070	0.18	0.42	0%	7.73	0.00	222.4	15.5
Madagascar	0.1	289	801	0.19	0.37	19%	6.57	0.09	1922.0	23.2
Mali	6.5	119	321	0.27	0.40	47%	3.49	−0.12	580.6	29.2
Mozambique	3.9	200	297	0.22	0.08	35%	10.63	0.04	289.8	26.0
Mauritania	0.1	37	102	0.21	1.39	6%	6.03	−0.14	208.7	29.9
Malawi	3.9	184	910	0.22	0.07	34%	2.35	0.04	237.7	23.3
Niger	2.1	76	347	0.86	0.44	27%	3.97	−0.02	482.2	29.3
Nigeria	3.8	203	300	0.22	0.13	34%	3.46	−0.09	1159.2	27.9
Rwanda	0.6	303	1772	0.15	0.11	5%	5.23	0.11	239.5	19.5
Senegal	12.2	102	31	0.23	0.25	5%	1.85	−0.17	254.1	28.2
South Sudan	0.0	196	516	0.27	1.43	17%	7.37	−0.06	2434.4	27.5
Tanzania	1.9	215	942	0.24	0.32	9%	8.32	0.08	1838.5	23.5
Uganda	2.3	300	1240	0.22	0.19	17%	5.73	0.04	316.4	22.6
South Africa	7.2	125	1102	0.31	0.28	2%	3.25	−0.01	2291.4	19.4
Zambia	6.3	171	1155	0.20	0.23	13%	7.27	0.06	541.8	22.6

**Table B3:** Summary of main weather shocks.

	Precipitation (flood)		Precipitation (drought)		Temperature (high)		SPEI (high)		SPEI (low)	
	1 s.d.	2 s.d.	1 s.d.	2 s.d.	1 s.d.	2 s.d.	1 s.d.	2 s.d.	1 s.d.	2 s.d.
Angola	0.1%	0.0%	0.0%	0.0%	42.0%	4.0%	0.0%	0.0%	0.0%	0.0%
Burkina Faso	0.0%	0.0%	0.0%	0.0%	1.2%	0.0%	26.9%	0.0%	0.0%	0.0%
Burundi	0.0%	0.0%	0.0%	0.0%	99.9%	19.2%	0.0%	0.0%	0.0%	0.0%
Cameroon	0.0%	0.0%	67.3%	0.0%	31.9%	0.0%	0.0%	0.0%	13.1%	0.0%
Cote d'Ivoire	0.0%	0.0%	0.0%	0.0%	0.0%	0.0%	0.0%	0.0%	0.0%	0.0%
DRC	6.1%	0.0%	0.0%	0.0%	1.2%	1.2%	0.0%	0.0%	0.0%	0.0%
Ethiopia	0.0%	0.0%	0.0%	0.0%	80.3%	2.6%	0.0%	0.0%	0.9%	0.0%
Ghana	0.0%	0.0%	13.7%	0.0%	10.8%	0.0%	1.6%	0.0%	3.5%	0.0%
Kenya	0.0%	0.0%	1.3%	0.0%	45.8%	1.5%	0.0%	0.0%	0.0%	0.0%
Lesotho	0.0%	0.0%	0.0%	0.0%	26.2%	0.0%	8.4%	0.0%	0.0%	0.0%
Madagascar	8.5%	0.0%	5.2%	0.0%	53.9%	0.0%	8.5%	0.0%	0.1%	0.0%
Malawi	0.0%	0.0%	17.6%	0.0%	82.6%	65.8%	0.0%	0.0%	0.0%	0.0%
Mali	41.3%	2.7%	0.0%	0.0%	4.6%	0.0%	0.0%	0.0%	0.0%	0.0%
Mauritania	51.9%	0.0%	0.0%	0.0%	5.4%	0.0%	0.0%	0.0%	4.1%	0.0%
Mozambique	0.0%	0.0%	0.2%	0.0%	22.7%	0.0%	0.6%	0.0%	5.5%	0.0%
Niger	0.9%	0.0%	0.0%	0.0%	0.0%	0.0%	2.5%	0.0%	0.0%	0.0%
Nigeria	19.1%	0.0%	1.2%	0.0%	5.8%	0.0%	32.7%	0.0%	26.0%	0.0%
Rwanda	0.0%	0.0%	23.5%	0.0%	18.0%	0.0%	0.0%	0.0%	0.0%	0.0%
Senegal	7.5%	0.0%	0.0%	0.0%	22.1%	0.0%	4.5%	0.0%	0.0%	0.0%
South Africa	0.0%	0.0%	0.0%	0.0%	52.4%	0.0%	11.7%	0.0%	0.0%	0.0%
South Sudan	0.0%	0.0%	0.0%	0.0%	39.2%	0.0%	30.0%	0.0%	0.0%	0.0%
Tanzania	0.0%	0.0%	1.8%	0.0%	11.0%	0.0%	0.0%	0.0%	0.0%	0.0%
Uganda	11.6%	0.0%	16.2%	0.0%	4.3%	0.0%	0.0%	0.0%	0.0%	0.0%
Zambia	0.0%	0.0%	0.0%	0.0%	41.8%	0.0%	0.0%	0.0%	0.0%	0.0%

**Overall**	**4.0%**	**0.1%**	**6.3%**	**0.0%**	**37.0%**	**4.4%**	**3.3%**	**0.0%**	**1.7%**	**0.0%**

**Table B4:** Intra-cluster correlations for country, rural/urban, region level.

	Intra-country	Intra-country and rural/urban	Intra-country, region and rural/urban
	interclass corr/(sd)	interclass corr/(sd)	interclass corr/(sd)
Tot. expenditure per capita (2011 PPP $)	0.124	0.173	0.190
	(0.038)	(0.036)	(0.013)
Food expenditure per capita (2011 PPP $)	0.073	0.107	0.132
	(0.026)	(0.027)	(0.011)
Poverty Rate 1.90$/day ($ 2011 PPP)	0.195	0.270	0.322
	(0.055)	(0.050)	(0.019)

**Table B5:** District level Moran I statistics of spatial correlation.

Variable	stat	mean	sd	z	p-value
Total expenditure per capita (2011 PPP $)	0.046	−0.001	0.002	24.955	0
Food expenditure per capita (2011 PPP $)	0.064	−0.001	0.002	29.356	0
Poverty Rate 1.90$/day ($ 2011 PPP)	0.24	−0.001	0.002	125.154	0
Rainfall average (1950-sv. year)	0.025	−0.001	0.002	13.477	0
Temperature average (1950-sv. year)	0.066	−0.001	0.002	35.162	0
SPEI average (1950-sv. year)	0.069	−0.001	0.002	37.122	0

**Table B6:** List of countries used in the regression analysis.

Country	Per capita total expenditure and poverty regressions	Per capita food expenditure regressions	Per capita exp. and poverty regressions with land owners interaction	Per capita food exp. regressions with land owners interaction
Angola	X	X	–	–
Burundi	X	X	X	X
Burkina Faso	X	X	X	X
Cote d'Ivoire	X	X	X	X
Cameroon	X	–	X	–
DRC	X	X	–	–
Ethiopia	X	X	–	–
Ghana	X	X	X	X
Kenya	X	X	X	X
Lesotho	X	–	X	–
Madagascar	X	X	X	X
Mali	X	–	–	–
Mozambique	X	X	X	X
Mauritania	X	X	X	X
Malawi	X	X	X	X
Niger	X	X	X	X
Nigeria	X	X	X	X
Rwanda	X	X	X	X
Senegal	X	X	X	X
South Sudan	X	X	X	X
Tanzania	X	X	X	X
Uganda	X	–	–	–
South Africa	X	X	–	–
Zambia	X	X	X	X

**Appendix C.1:** Household level OLS complete regression: SPEI shock.

	Log per capita expenditure ($ 2011 PPP)		Food per capita expenditure ($ 2011 PPP)		Poverty Rate 1.90$/day ($ 2011 PPP)	
	(1)	(2)	(3)	(4)	(5)	(6)
	coef/se	coef/se	coef/se	coef/se	coef/se	coef/se
SPEI, long term average	1.261***	1.222***	1.392***	1.374***	−0.593***	−0.596***
	(0.134)	(0.141)	(0.138)	(0.143)	(0.072)	(0.075)
SPEI flood shock dummy (1 sd)	−0.197***	−0.195***	−0.171***	−0.185***	0.135***	0.134***
	(0.052)	(0.057)	(0.052)	(0.059)	(0.030)	(0.031)
SPEI drought shock dummy (1 sd)	0.012	0.060	0.002	0.026	−0.025	−0.038
	(0.050)	(0.050)	(0.055)	(0.054)	(0.031)	(0.031)
Household is rural	−0.395***	−0.354***	−0.238***	−0.226***	0.178***	0.168***
	(0.018)	(0.019)	(0.018)	(0.019)	(0.010)	(0.011)
Household head is female	−0.069***	−0.072***	−0.045***	−0.046***	0.008	0.009
	(0.015)	(0.014)	(0.011)	(0.011)	(0.006)	(0.006)
Age of household head	−0.002***	−0.002***	−0.004***	−0.004***	0.001***	0.001***
	(0.000)	(0.000)	(0.000)	(0.000)	(0.000)	(0.000)
Mean level of educ of adults (>15) in the household	0.060***	0.058***	0.034***	0.033***	−0.023***	−0.022***
	(0.003)	(0.003)	(0.002)	(0.002)	(0.001)	(0.001)
Share of working age in the household	0.365***	0.352***	0.234***	0.229***	−0.166***	−0.163***
	(0.024)	(0.024)	(0.025)	(0.025)	(0.014)	(0.013)
Number of children (<6) in the household	−0.094***	−0.093***	−0.102***	−0.102***	0.046***	0.046***
	(0.005)	(0.004)	(0.005)	(0.005)	(0.002)	(0.002)
Nighlight		0.007***		0.003***		−0.002***
		(0.001)		(0.001)		(0.000)
Growing period (days, mean)		−0.000		−0.000		0.000
		(0.000)		(0.000)		(0.000)
Elevation (meter, mean)		−0.000		−0.000		0.000
		(0.000)		(0.000)		(0.000)
Per capita crop land area in the district (Ha)		0.051***		0.070***		−0.034***
		(0.017)		(0.018)		(0.010)
Per capita total livestock units in the district (Ha)		−0.025**		−0.035***		0.011*
		(0.011)		(0.011)		(0.006)
Time to 20 k market (hrs, median)		0.001		0.001		−0.001*
		(0.001)		(0.001)		(0.001)
Constant	3.894***	3.928***	4.075***	4.352***	0.532***	−0.064
	(0.050)	(0.065)	(0.048)	(0.120)	(0.025)	(0.065)

Number of observations	2,50,272	2,49,876	2,19,429	2,19,033	2,50,396	2,50,000
N pop	70,01,66,623	69,97,91,188	62,90,58,208	62,86,82,773	70,03,14,521	69,99,39,086
N strata	24	24	20	20	24	24
R2	0.464	0.470	0.265	0.267	0.280	0.281

Note: 0.01 – ***; 0.05 – **; 0.1 – *.

Standard errors clustered at the region level. Country, year, and month fixed effects included but not reported. SPEI is an index of evapotranspiration that combines temperature and humidity, with a high level of SPEI identifying humid and hot areas. Flood shock is a dummy that equals one if the value of SPEI for the planting and growing season preceding the survey is above 1 SD from the long term average (for the same months in the same region). Drought shocks are identified the same way except that the value is higher than 1 SD below the long term average. For each outcome, the first specification controls only for demographic characteristics of the household while the second adds a series of geographic controls as well as an asset index constructed using a principal component analysis on a set of dwelling indicators (access to electricity; ownership of television, radio, motorbike, telephone, fridge, bicycle, car, draft animals, house; access to running water, toilet; use of concrete roof, concrete or brick walls, concrete floor). The countries included in regressions on total expenditure per capita and on poverty rate are Angola, Burundi, Burkina Faso, Cote d'Ivoire, Cameroon, DRC, Ethiopia, Ghana, Kenya, Lesotho, Madagascar, Mali, Mozambique, Mauritania, Malawi, Niger, Nigeria, Rwanda, Senegal, South Sudan, Tanzania, Uganda, South Africa and Zambia. Regressions on food expenditure per capita exclude Uganda, Mali, Lesotho and Cameroon because of lack of officially calculated food data from the household surveys.

**Appendix C.2:** Household level OLS regressions evaluating effect of adding asset index control.

	Log per capita expenditure ($ 2011 PPP)		Food per capita expenditure ($ 2011 PPP)		Poverty Rate 1.90$/day ($ 2011 PPP)	
	(1)	(2)	(3)	(4)	(5)	(6)
	coef/se	coef/se	coef/se	coef/se	coef/se	coef/se
*Panel A: SPEI measure of weather*						
SPEI, long term average	1.222***	1.201***	1.374***	1.451***	−0.596***	−0.574***
	(0.141)	(0.150)	(0.143)	(0.151)	(0.075)	(0.082)
SPEI flood shock dummy (1 sd)	−0.195***	−0.136**	−0.185***	−0.153***	0.134***	0.103***
	(0.057)	(0.056)	(0.059)	(0.059)	(0.031)	(0.030)
SPEI drought shock dummy (1 sd)	0.060	0.028	0.026	0.003	−0.038	−0.023
	(0.050)	(0.050)	(0.054)	(0.054)	(0.031)	(0.031)
Asset index		0.187***		0.113***		−0.077***
		(0.007)		(0.006)		(0.003)

Number of observations	2,49,876	2,22,085	2,19,033	1,91,242	2,50,000	2,22,209
N pop	69,97,91,188	62,37,61,214	62,86,82,773	55,26,52,799	69,99,39,086	62,39,09,112
N strata	24	23	20	19	24	23
R2	0.470	0.532	0.267	0.302	0.281	0.328

*Panel B: rainfall and temperature measures of weather*						
Rainfall long term average (dm)	−0.001	0.001	0.000	0.001	−0.001**	−0.002***
	(0.001)	(0.001)	(0.001)	(0.001)	(0.000)	(0.000)
Temperature long term average (C)	−0.046***	−0.054***	−0.046***	−0.059***	0.028***	0.035***
	(0.006)	(0.006)	(0.006)	(0.007)	(0.003)	(0.003)
Rainfall flood shock dummy (1 sd)	−0.354***	−0.269***	−0.340***	−0.268***	0.167***	0.126***
	(0.047)	(0.048)	(0.056)	(0.057)	(0.026)	(0.025)
Rainfall drought shock dummy (1 sd)	0.164***	0.096**	0.167***	0.125**	−0.103***	−0.069***
	(0.040)	(0.039)	(0.051)	(0.050)	(0.023)	(0.023)
Temperature heat shock dummy (2 sd)	0.124*	0.160***	0.135*	0.193***	−0.012	−0.041
	(0.065)	(0.058)	(0.071)	(0.062)	(0.038)	(0.035)
Asset index		0.181***		0.107***		−0.075***
		(0.007)		(0.006)		(0.003)

Number of observations	2,50,552	2,22,761	2,19,692	1,91,901	2,50,676	2,22,885
N pop	70,12,73,629	62,52,43,655	63,00,51,532	55,40,21,558	70,14,21,527	62,53,91,552
N strata	24	23	20	19	24	23
R2	0.475	0.536	0.271	0.305	0.285	0.332

Note: 0.01 – ***; 0.05 – **; 0.1 – *.

This set of regressions exclude Ethiopia -for which asset information is unavailable- and compared the main results on the other countries with and without asset control. Standard errors are clustered at the region level. Country, year, and month fixed effects as well as socio-demographic and geographic controls are included but not reported. Panel A identifies shocks using SPEI, while panel B identifies shocks using temperature and rainfall. SPEI is an index of evapotranspiration that combines temperature and humidity, with a high level of SPEI identifying hot and humid areas. Flood shock is a dummy that equals one if the value of SPEI for the planting and growing season preceding the survey is above 1 SD from the long term average (for the same months in the same region). Drought shocks are identified the same way except that the value needs to be higher than 1 SD below the long term average. In panel B flood/drought dummy equal 1 when rainfall in the previous season is 1 SD above/below the long term average, while heat shock equal 1 when the temperature is 2 SD above the long term average. Countries included in regressions on per-capita total expenditure and on poverty headcount ratio are Angola, Burundi, Burkina Faso, Cote d'Ivoire, Cameroon, DRC, Ghana, Kenya, Lesotho, Madagascar, Mali, Mozambique, Mauritania, Malawi, Niger, Nigeria, Rwanda, Senegal, South Sudan, Tanzania, Uganda, South Africa and Zambia. Regressions on food expenditure per capita exclude Uganda, Mali, Lesotho and Cameroon because of lack of officially calculated food data from the household surveys.

**Appendix C.3:** Household level OLS regressions evaluating effect of adding malaria incidence control.

	Log per capita expenditure ($ 2011 PPP)			Food per capita expenditure ($ 2011 PPP)			Poverty Rate 1.90$/day ($ 2011 PPP)		
	(1)	(2)	(3)	(4)	(5)	(6)	(7)	(8)	(9)
	coef/se	coef/se	coef/se	coef/se	coef/se	coef/se	coef/se	coef/se	coef/se
Rainfall long term average (dm)	−0.001	−0.001	−0.001	0.000	−0.001	−0.000	−0.001**	−0.001*	−0.001*
	(0.001)	(0.001)	(0.001)	(0.001)	(0.001)	(0.001)	(0.000)	(0.000)	(0.000)
Temperature long term average (C)	−0.046***	−0.046***	−0.039***	−0.046***	−0.047***	−0.039***	0.028***	0.028***	0.021***
	(0.006)	(0.006)	(0.006)	(0.006)	(0.006)	(0.006)	(0.003)	(0.003)	(0.003)
Rainfall flood shock dummy (1 sd)	−0.354***	−0.357***	−0.363***	−0.340***	−0.345***	−0.351***	0.167***	0.168***	0.175***
	(0.047)	(0.047)	(0.047)	(0.056)	(0.057)	(0.057)	(0.026)	(0.026)	(0.025)
Rainfall drought shock dummy (1 sd)	0.164***	0.131***	0.124***	0.167***	0.118**	0.122**	−0.103***	−0.089***	−0.082***
	(0.040)	(0.039)	(0.039)	(0.051)	(0.048)	(0.048)	(0.023)	(0.023)	(0.023)
Temperature heat shock dummy (2 sd)	0.124*	0.140**	0.134*	0.135*	0.157**	0.149**	−0.012	−0.019	−0.013
	(0.065)	(0.067)	(0.069)	(0.071)	(0.074)	(0.076)	(0.038)	(0.039)	(0.040)
Malaria incidence		0.359***	1.576***		0.507***	2.425***		−0.160***	−1.409***
		(0.080)	(0.418)		(0.088)	(0.582)		(0.046)	(0.231)
Temperature (LT) * Malaria			−0.048***			−0.076***			0.049***
			(0.017)			(0.024)			(0.010)

Number of observations	2,50,552	2,50,552	2,50,552	2,19,692	2,19,692	2,19,692	2,50,676	2,50,676	2,50,676
N_pop	70,12,73,629	70,12,73,629	70,12,73,629	63,00,51,532	63,00,51,532	63,00,51,532	70,14,21,527	70,14,21,527	70,14,21,527
N_strata	24	24	24	20	20	20	24	24	24
R2	0.475	0.477	0.477	0.271	0.274	0.275	0.285	0.286	0.287

Note: 0.01 – ***; 0.05 – **; 0.1 – *.

Standard errors clustered at region level. Country, year, and month fixed effects included but not reported. In this table we test the effect of adding malaria incidence at the district-level as additional control. We only report the regressions using temperature and rainfall, as SPEI is likely to be highly collinear with malaria. The flood/drought dummy equals 1 when rainfall in the previous season is 1 SD above/below the long term average, while heat shock equal 1 when temperature is 2 SD above the long term average. For each outcome, the first column presents the main specification used in the analysis which excludes malaria; the second adds malaria incidence as additional control; and the third includes a further interaction between malaria and long term temperature. Countries included in the regressions on per-capita total expenditure and on poverty headcount ratios are Angola, Burundi, Burkina Faso, Cote d'Ivoire, Cameroon, DRC, Ethiopia, Ghana, Kenya, Lesotho, Madagascar, Mali, Mozambique, Mauritania, Malawi, Niger, Nigeria, Rwanda, Senegal, South Sudan, Tanzania, Uganda, South Africa and Zambia. Regressions on food expenditure per capita exclude Uganda, Mali, Lesotho and Cameroon because of lack of officially calculated food data from the household surveys.

**Appendix C.4:** Household level OLS regressions for comparable samples across outcomes.

	Overall			Rural only		
	Log per capita expenditure ($ 2011 PPP)	Food per capita expenditure ($ 2011 PPP)	Poverty Rate 1.90$/day ($ 2011 PPP)	Log per capita expenditure ($ 2011 PPP)	Food per capita expenditure ($ 2011 PPP)	Poverty Rate 1.90$/day ($ 2011 PPP)
	(1)	(2)	(3)	(4)	(5)	(6)
	coef/se	coef/se	coef/se	coef/se	coef/se	coef/se
*Panel A: Using SPEI shocks*						
SPEI, long term average	1.148***	1.374***	−0.530***	1.409***	1.557***	−0.755***
	(0.152)	(0.143)	(0.082)	(0.170)	(0.178)	(0.096)
SPEI flood shock dummy (1 sd)	−0.202***	−0.185***	0.147***	−0.203***	−0.184**	0.134***
	(0.059)	(0.059)	(0.032)	(0.078)	(0.081)	(0.041)
SPEI drought shock dummy (1 sd)	0.083	0.026	−0.057*	−0.073	−0.135*	0.042
	(0.054)	(0.054)	(0.033)	(0.078)	(0.078)	(0.045)

Controls	ALL	ALL	ALL	ALL	ALL	ALL
Number of observations	2,19,780	2,19,033	2,19,904	1,16,027	1,15,734	1,16,033
N_pop	62,95,25,809	62,86,82,773	62,96,73,707	41,98,58,620	41,95,72,391	41,98,63,783
N_strata	20	20	20	20	20	20
R2	0.473	0.267	0.285	0.339	0.204	0.216

*Panel B: Using temperature and rain shocks*						
Rainfall long term average (dm)	−0.000	0.000	−0.001**	0.002**	0.002	−0.002***
	(0.001)	(0.001)	(0.000)	(0.001)	(0.001)	(0.001)
Temperature long term average (C)	−0.052***	−0.046***	0.032***	−0.040***	−0.038***	0.022***
	(0.007)	(0.006)	(0.004)	(0.006)	(0.007)	(0.004)
Rainfall flood shock dummy (1 sd)	−0.370***	−0.340***	0.183***	−0.343***	−0.334***	0.160***
	(0.058)	(0.056)	(0.031)	(0.067)	(0.069)	(0.036)
Rainfall drought shock dummy (1 sd)	0.208***	0.167***	−0.130***	0.071	0.062	−0.055
	(0.062)	(0.051)	(0.035)	(0.073)	(0.068)	(0.055)
Temperature heat shock dummy (2 sd)	0.082	0.135*	0.009	0.124**	0.150**	−0.017
	(0.059)	(0.071)	(0.037)	(0.061)	(0.067)	(0.038)

Controls	ALL	ALL	ALL	ALL	ALL	ALL
Number of observations	2,20,439	2,19,692	2,20,563	1,16,519	1,16,226	1,16,525
N_pop	63,08,94,568	63,00,51,532	63,10,42,465	42,06,90,293	42,04,04,064	42,06,95,455
N_strata	20	20	20	20	20	20
R2	0.479	0.271	0.290	0.344	0.206	0.217

Note: 0.01 – ***; 0.05 – **; 0.1 – *.

**Appendix C.5:** Robustness of household level OLS regressions to different shock measures.

	LT average and shock since 1990			Shock using deviation from median since 1950		
	Log per capita expenditure ($ 2011 PPP)	Food per capita expenditure ($ 2011 PPP)	Poverty Rate 1.90$/day ($ 2011 PPP)	Log per capita expenditure ($ 2011 PPP)	Food per capita expenditure ($ 2011 PPP)	Poverty Rate 1.90$/day ($ 2011 PPP)
	(1)	(2)	(3)	(4)	(5)	(6)
	coef/se	coef/se	coef/se	coef/se	coef/se	coef/se
*Panel A: Using SPEI shocks*						
SPEI, long term average/median	0.440***	0.525***	−0.220***	0.830***	0.914***	−0.448***
	(0.080)	(0.082)	(0.046)	(0.089)	(0.100)	(0.056)
SPEI flood shock dummy (1 sd)	−0.128***	−0.105**	0.091***	−0.150***	−0.122**	0.112***
	(0.041)	(0.043)	(0.022)	(0.048)	(0.050)	(0.025)
SPEI drought shock dummy (1 sd)	0.195***	0.200***	−0.100***	−0.059	−0.083	0.048*
	(0.049)	(0.049)	(0.031)	(0.046)	(0.051)	(0.029)

Controls	ALL	ALL	ALL	ALL	ALL	ALL
Number of observations	2,49,876	2,19,033	2,50,000	2,49,876	2,19,033	2,50,000
N_pop	69,97,91,188	62,86,82,773	69,99,39,086	69,97,91,188.413	62,86,82,773.442	69,99,39,086.214
N_strata	24	20	24	24.000	20.000	24.000
R2	0.467	0.263	0.279	0.468	0.264	0.280

*Panel B: Using temperature and rain shocks*						
Rainfall long term average / median (dm)	−0.000*	−0.000	−0.000	−0.000*	−0.000	−0.000
	(0.000)	(0.000)	(0.000)	(0.000)	(0.000)	(0.000)
Temperature long term average / median (C)	−0.042***	−0.042***	0.026***	−0.044***	−0.044***	0.027***
	(0.005)	(0.006)	(0.003)	(0.006)	(0.006)	(0.003)
Rainfall flood shock dummy (1 sd)	−0.249***	−0.195***	0.117***	−0.241***	−0.189***	0.109***
	(0.040)	(0.047)	(0.022)	(0.040)	(0.047)	(0.022)
Rainfall drought shock dummy (1 sd)	−0.044	−0.097**	0.005	−0.024	−0.048	0.003
	(0.029)	(0.041)	(0.018)	(0.031)	(0.039)	(0.020)
Temperature heat shock dummy (2 sd)	−0.439***	−0.311**	0.201***	0.043	−0.010	0.023
	(0.114)	(0.124)	(0.049)	(0.054)	(0.051)	(0.035)

Controls	ALL	ALL	ALL	ALL	ALL	ALL
Number of observations	2,50,552	2,19,692	2,50,676	2,50,552	2,19,692	2,50,676
N_pop	70,12,73,629	63,00,51,532	70,14,21,527	70,12,73,628.758	63,00,51,531.787	70,14,21,526.559
N_strata	24	20	24	24.000	20.000	24.000
R2	0.471	0.264	0.282	0.471	0.264	0.282

Note: 0.01 – ***; 0.05 – **; 0.1 – *.

Standard errors are clustered at the region level. Country, year, and month fixed effects as well as socio-demographic and geograhic controls are included but not reported. In Panel A shocks is identified using SPEI while in panel B shocks are based on temperature and rainfall information. SPEI is an index of evapotranspiration that combines temperature and humidity, with a high level of SPEI identifying hot and humid areas. Column (1) to (3) consider long term averages in climatic conditions over the period from 1990 up to the year of the survey -while in main specification the period considered starts in 1950- and compute the shocks as dummies for more than 1 or 2 SD differences from long term averages. Column (4) to (6) report long term medians in climatic conditions from the 1950 up to the year of the survey and compute the shocks as dummies for more than 1 or 2 SD differences from the long term median. Countries included in the regressions on per-capita total expenditure and on poverty headcount ratios are Angola, Burundi, Burkina Faso, Cote d'Ivoire, Cameroon, DRC, Ethiopia, Ghana, Kenya, Lesotho, Madagascar, Mali, Mozambique, Mauritania, Malawi, Niger, Nigeria, Rwanda, Senegal, South Sudan, Tanzania, Uganda, South Africa, and Zambia. Regressions on per-capita food expenditure exclude Uganda, Mali, Lesotho and Cameroon because of lack of officially calculated food data from the household surveys.

**Appendix D.1:** Spatial District level complete regression: SPEI differences from long term mean.

	Log per capita expenditure ($ 2011 PPP)		Food per capita expenditure ($ 2011 PPP)		Poverty Rate 1.90$/day ($ 2011 PPP)	
	(1)	(2)	(3)	(4)	(5)	(6)
	coef/se	coef/se	coef/se	coef/se	coef/se	coef/se
SPEI, long term average	1.943***	1.855***	2.061***	1.973***	−0.916***	−0.879***
	(0.134)	(0.134)	(0.133)	(0.128)	(0.079)	(0.076)
SPEI flood shock dummy (1 sd)	−0.433***	−0.463***	−0.455***	−0.515***	0.239***	0.260***
	(0.034)	(0.036)	(0.035)	(0.035)	(0.020)	(0.020)
SPEI drought shock dummy (1 sd)	0.294***	0.318***	0.175***	0.207***	−0.110***	−0.122***
	(0.029)	(0.028)	(0.029)	(0.028)	(0.017)	(0.016)
Spatially lagged SPEI LT average	3.214***	4.285***	9.038***	10.967***	−3.709***	−4.270***
	(1.233)	(1.211)	(1.315)	(1.228)	(0.729)	(0.674)
Share of rural households	−0.504***	−0.540***	−0.547***	−0.551***	0.330***	0.338***
	(0.040)	(0.040)	(0.041)	(0.040)	(0.023)	(0.023)
Share of female headed households	−1.402***	−1.503***	−0.421***	−0.683***	−0.000	0.182**
	(0.130)	(0.145)	(0.129)	(0.140)	(0.076)	(0.082)
Average age of household heads	−0.032***	−0.027***	−0.031***	−0.030***	0.016***	0.018***
	(0.004)	(0.004)	(0.004)	(0.004)	(0.002)	(0.002)
Mean level of educ of adults (>15)	0.108***	0.126***	0.037***	0.072***	−0.040***	−0.063***
	(0.007)	(0.007)	(0.007)	(0.007)	(0.004)	(0.004)
Share of working age in the district	1.737***	1.079***	0.387	−0.116	−0.362**	−0.162
	(0.246)	(0.253)	(0.255)	(0.249)	(0.145)	(0.142)
Average N. of children (<6) per household	−0.148***	−0.144***	−0.168***	−0.172***	0.085***	0.108***
	(0.031)	(0.031)	(0.035)	(0.033)	(0.018)	(0.017)
Nighlight		0.005***		0.001		0.002*
		(0.001)		(0.001)		(0.001)
Growing period (days, mean)		0.000**		0.000		−0.000
		(0.000)		(0.000)		(0.000)
Elevation (meter, mean)		0.000		−0.000**		0.000**
		(0.000)		(0.000)		(0.000)
Per capita crop land area in the district (Ha)		0.581***		0.650***		−0.347***
		(0.055)		(0.053)		(0.031)
Per capita TLU in the district (Ha)		0.018		0.134**		−0.132***
		(0.064)		(0.061)		(0.036)
Time to 20 k market (hrs, median)		0.005*		0.004		−0.004***
		(0.003)		(0.003)		(0.002)

Constant	40,738.491	28,966.749	1,00,358.835	31,333.929	−78,639.636	−24,039.783
	(46,199.919)	(61,281.792)	(1,03,259.112)	(1,19,334.149)	(77,088.852)	(66,547.677)

Lambda	0.830***	0.881***	0.923***	0.943***	0.937***	0.941***
	(0.121)	(0.087)	(0.055)	(0.042)	(0.045)	(0.043)
Sigma	2,93,103.373***	2,80,375.346***	2,87,946.237***	2,63,329.186***	1,72,508.376***	1,57,799.459***
	(4,844.188)	(4,633.376)	(5,078.132)	(4,643.375)	(2,849.727)	(2,606.730)
Number of observations	1,838	1,838	1,614	1,614	1,838	1,838
Log-Likelihood	−25,749.42	−25,668.78	−22,585.40	−22,441.89	−24,777.66	−24,614.01
chi2	3,99,809.106	4,35,237.883	2,66,059.246	3,17,580.525	16,181.322	19,694.098
p	0.002	0.000	0.000	0.000	0.000	0.000
Wald	47.374	103.257	277.200	514.182	426.656	483.513

Note: 0.01 – ***; 0.05 – **; 0.1 – *.

Spatial error correction model with spatially lagged long term SPEI average added as control. Country, year, and month fixed effects are included but not reported. SPEI is an index of evapotranspiration that combines temperature and humidity, with a high level of SPEI identifying humid and hot areas. Flood shock is a dummy that equals one if the value of SPEI for the planting and growing season preceding the survey is above 1 SD from the long term average -for the same months and region. Drought shocks are identified the same way except that the value is higher than 1 SD below the long term average. For each outcome, in the first specification estimates only control for average socio-demographic characteristics of the district, while in the second adds they control also for a series of geographic controls. Countries included in the regressions on per capita total expenditure and on poverty headcount ratios are Angola, Burundi, Burkina Faso, Cote d'Ivoire, Cameroon, DRC, Ethiopia, Ghana, Kenya, Lesotho, Madagascar, Mali, Mozambique, Mauritania, Malawi, Niger, Nigeria, Rwanda, Senegal, South Sudan, Tanzania, Uganda, South Africa and Zambia. Regressions on per-capita food expenditure exclude Uganda, Mali, Lesotho and Cameroon because of lack of officially calculated food data from the household surveys.

**Appendix D.2:** Spatial district level regression: coefficients of interest on the main shock measures.

	Log per capita expenditure ($ 2011 PPP)	Food per capita expenditure ($ 2011 PPP)	Poverty Rate 1.90$/day ($ 2011 PPP)
	(1)	(2)	(3)
	coef/se	coef/se	coef/se
*Panel A: Using SPEI shocks*			
SPEI, long term average	1.855***	1.973***	−0.879***
	(0.134)	(0.128)	(0.076)
SPEI flood shock dummy (1 sd)	−0.463***	−0.515***	0.260***
	(0.036)	(0.035)	(0.020)
SPEI drought shock dummy (1 sd)	0.318***	0.207***	−0.122***
	(0.028)	(0.028)	(0.016)

Controls	ALL	ALL	ALL
Number of observations	1,838	1,614	1,838
Log-Likelihood	−25,668.78	−22,441.89	−24,614.01
chi2	4,35,237.883	3,17,580.525	19,694.098
p	0.000	0.000	0.000
Wald	103.257	514.182	483.513

*Panel B: Using temperature and rain shocks*			
Rainfall long term average (dm)	0.000	0.001*	−0.002***
	(0.001)	(0.001)	(0.000)
Temperature long term average (C)	−0.073***	−0.076***	0.040***
	(0.008)	(0.008)	(0.005)
Rainfall flood shock dummy (1 sd)	−0.624***	−0.535***	0.270***
	(0.028)	(0.028)	(0.016)
Rainfall drought shock dummy (1 sd)	−0.172	0.074	0.167**
	(0.121)	(0.396)	(0.069)
Temperature heat shock dummy (2 sd)	0.264	0.096	0.035
	(0.495)	(0.489)	(0.282)

Controls	ALL	ALL	ALL
Number of observations	1838	1614	1838
Log-Likelihood	−25,711.66	−22,549.92	−24,675.94
chi2	4,16,696.081	2,78,440.582	18,324.967
p	0.000	0.000	0.000
Wald	52.139	184.015	194.605

Note: 0.01 – ***; 0.05 – **; 0.1 – *.

Spatial error correction model with spatially lagged long term SPEI average (panel A), and long term rainfall and temperature average (panel B) added as control. Country, year, and month fixed effects included but not reported. SPEI is an index of evapotranspiration that combines temperature and humidity, with a high level of SPEI identifying humid and hot areas. Flood shock is a dummy that equals one if the value of SPEI for the planting and growing season preceding the survey is above 1 SD from the long term average (for the same months in the same region). Drought shocks are identified the same way except that the value is higher than 1 SD below the long term average. In Panel B shocks are identified using information on rainfall and temperature. Here the flood/drought dummy equal 1 when rainfall in the previous season is 1 SD above/below the long term average, while heat shock equal 1 when the temperature is 2 SD above the long term average. Countries included in the regressions on per capita total expenditure and on poverty headcount ratios are Angola, Burundi, Burkina Faso, Cote d'Ivoire, Cameroon, DRC, Ethiopia, Ghana, Kenya, Lesotho, Madagascar, Mali, Mozambique, Mauritania, Malawi, Niger, Nigeria, Rwanda, Senegal, South Sudan, Tanzania, Uganda, South Africa and Zambia. Regressions on per-capita food expenditure exclude Uganda, Mali, Lesotho and Cameroon because of lack of officially calculated food data from the household surveys.

**Appendix E:** Household level OLS regressions with smallholder and large-holder farmers interactions.

	Log per capita expenditure ($ 2011 PPP)	Food per capita expenditure ($ 2011 PPP)	Poverty Rate 1.90$/day ($ 2011 PPP)
	(1)	(2)	(3)
*SPEI shocks*	coef/se	coef/se	coef/se
Omitted category = non farming HH			
Smallholder	−0.216***	−0.151***	0.121***
	(0.019)	(0.019)	(0.011)
Largeholder	−0.116***	−0.053**	0.082***
	(0.020)	(0.021)	(0.012)
SPEI, long term average	0.977***	1.284***	−0.568***
	(0.180)	(0.191)	(0.099)
Flood shock dummy (1 sd)	0.205***	0.263***	−0.048*
	(0.056)	(0.057)	(0.028)
Drought shock dummy (1 sd)	−0.008	−0.154*	0.024
	(0.056)	(0.091)	(0.029)
Smallholder * flood shock	−0.393***	−0.449***	0.195***
	(0.083)	(0.086)	(0.043)
Smallholder * drought shock	−0.067	0.028	0.013
	(0.090)	(0.119)	(0.047)
Largeholder * flood shock	−0.193**	−0.282***	0.034
	(0.085)	(0.085)	(0.057)
Largeholder * drought shock	0.157**	0.382***	−0.088*
	(0.077)	(0.114)	(0.047)

Controls	ALL	ALL	ALL
Number of observations	1,32,437	1,14,979	1,32,558
N_pop	33,77,99,018	31,81,73,408	33,79,44,561
N_strata	18	16	18
R2	0.377	0.233	0.243

Note: 0.01 – ***; 0.05 – **; 0.1 – *.

Standard errors clustered at the region level. Country, year, and month fixed effects as well as socio-demographic and geographic controls included but not reported. *Smallholder* and 1 *Largeholder* are dummies identifying farming households with land size below or above 2ha, respectively. The omitted category is non-farming households. SPEI is an index of evapotranspiration that combines temperature and humidity, with a high level of SPEI identifying hot and humid areas. Flood shock is a dummy that equals one if the value of SPEI for the planting and growing season preceding the survey is above 1 SD from the long term average (for the same months and region). Drought shocks are identified the same way except that the value is higher than 1 SD below the long term average. Countries included in the regressions on per-capita total expenditure and on poverty headcount ratios are Burundi, Burkina Faso, Cote d'Ivoire, Cameroon, Ghana, Kenya, Lesotho, Madagascar, Mozambique, Mauritania, Malawi, Niger, Nigeria, Rwanda, Senegal, South Sudan, Tanzania and Zambia. Regressions on per-capita food expenditure exclude Lesotho and Cameroon because of lack of officially calculated food data from the household surveys.

## Appendix B. Summary statistics

Table B.1 highlights that, except from South Africa, all the other countries in our sample are prevalently rural, and show low levels of female headship (below 30%) as well as educational attainment (about 4 years of education on average), although with some heterogeneity across them. Finally, in most countries households have more than one child below 6 years old on average, signaling a relatively high share of children in the population. For district-level characteristics (Table B.2), the highest concentration of nightlight is found in Senegal, Ghana, and Angola; while the lowest in Mauritania, Madagascar, and DRC. Rwanda and Ethiopia show the lowest rates of cropland per capita, while Burkina Faso the highest. The latter also reports the highest rate of TLU per capita, while Burundi and Malawi the lowest.

Table B.3 shows the country-level incidence of climate shocks of various intensities (high and low SPEI, rainfall, and temperature shocks at one, two, and three standard deviations). For SPEI and rainfall, it is not uncommon for sampled households to experience differences of one standard deviation from the 1950 average. However, almost no households experienced annual deviations more extreme than one standard deviation. Yearly temperature shocks are more common- 37% percent of households experienced temperature at least one standard deviation above the mean, while 4.4% of households experienced temperature shocks at least two standard deviations above the mean. For our analysis, we define shocks as strictly as possible while ensuring an adequate number of households experiencing it. Therefore, we examine SPEI and rainfall shocks above and below one standard deviation, and temperature shocks above two standard deviations.

## Footnotes

1 The PPP relies on construction of an adjusted exchange rate for each country that equalizes the nominal exchange rate in terms of the local cost of a common basket of goods and services. In this exercise, monetary values in all countries in different years have been converted to international dollar at the 2011 PPP conversion rate (see World Bank, 2015).

2 Despite the heavy reliance of the region on rainfed agriculture (Kotir, 2011), the specific characteristics of farming systems and the adoption of drought-tolerant crop varieties might mitigate agriculture losses due to extreme precipitation shortage.

3 Expressed as the number of units in the universe -or reference- population each observation represents, with its selection based on the inverse of the probability of inclusion in the sample given the sampling design.

4 http://iresearch.worldbank.org/PovcalNet/index.htm.

5 In this study, we do not use regional spatial deflators within countries as they might potentially harm comparability across countries, rendering the resulting poverty figures not comparable with official statistics, and plausibly confusing the readers. While this decision implies that shock-prone areas might face higher prices affecting location-specific consumption expenditure values, the net effect might be ambiguous, depending on the marketed production share of the households affected (that is, whether they are net buyers versus net-sellers). Moreover, we did not want to make any assumptions about the -usually controversial- spatial deflation to be used.

6 Only few surveys (provided by the LSMS-ISA project, see http://go.worldbank.org/BCLXW38HY0) in our dataset are georeferenced.

7 As input data, the most recent SPEI dataset for 1901–2014 uses the Climatic Research Unit (University of East Anglia) Time-Series v.3.23 dataset (see Vicente-Serrano, 2010).

8 We limit the months of planting and growing seasons to maize only, as given the high number of countries and the potentially different main crop for each country and picking up a different period by country would have rendered the interpretation of our multivariate results potentially more confusing, based on different time periods. We picked maize as it is the most common staple across many agriculture-based farming systems across sub-Saharan Africa. FAO data on maize cropping season are retrieved from http://www.fao.org/agriculture/seed/cropcalendar/welcome.do.

9 Despite the binary nature of the outcome, poverty regressions at the household level are estimated using a linear probability model in order to be consistent with the district-level regressions, the latter estimated on average poverty rates (a continuous -not binary- measure at the district-level). Coefficients in both cases are to be interpreted as percentage points changes in poverty rates.

10 Maps based on other poverty measures are not reported for reason of space, but they are available from the authors upon request.

11 This index is constructed using factor analysis (principal-component factor method) based on the predicted value of the first factor in the principal component based on housing/dwelling conditions, number of asset durables (agricultural and non-agricultural), livestock, as well as land ownership. Weights assigned to each variable in the index are obtained according to the methodology proposed by Filmer and Pritchett (2001).

12 Despite the negative bivariate correlation between malaria incidence and expenditure, the multivariate regression coefficient shows a surprising, positive sign. The latter is due to the fact that the effect is mediated by temperature: the third column for each outcome shows that malaria is negatively associated with welfare in hotter areas, as previously shown by the literature (Frank et al., 2003; Kibret et al., 2016; Rogers & Randolph, 2000; Utzinger et al., 2005). Given that the malaria spatial layer is partly computed using temperature and rainfall, we excluded the variable from the main analysis to avoid potential multicollinearity.

13 Estimates by agro-ecology are not reported for reason of space, but available from the authors upon request.
